# Analysis of knockout mice suggests a role for VGF in the control of fat storage and energy expenditure

**DOI:** 10.1186/1472-6793-9-19

**Published:** 2009-10-28

**Authors:** Elizabeth Watson, Samira Fargali, Haruka Okamoto, Masato Sadahiro, Ronald E Gordon, Tandra Chakraborty, Mark W Sleeman, Stephen R Salton

**Affiliations:** 1 Department of Neuroscience, Mount Sinai School of Medicine, New York, NY, USA; 2 Regeneron Pharmaceuticals Inc., Tarrytown, NY, USA; 3 Department of Pathology, Mount Sinai School of Medicine, New York, NY, USA; 4 Biology Department, Adelphi University, Garden City, NY, USA; 5 Department of Geriatrics, Mount Sinai School of Medicine, New York, NY, USA

## Abstract

**Background:**

Previous studies of mixed background mice have demonstrated that targeted deletion of *Vgf *produces a lean, hypermetabolic mouse that is resistant to diet-, lesion-, and genetically-induced obesity. To investigate potential mechanism(s) and site(s) of action of VGF, a neuronal and endocrine secreted protein and neuropeptide precursor, we further analyzed the metabolic phenotypes of two independent VGF knockout lines on C57Bl6 backgrounds.

**Results:**

Unlike hyperactive VGF knockout mice on a mixed C57Bl6-129/SvJ background, homozygous mutant mice on a C57Bl6 background were hypermetabolic with similar locomotor activity levels to *Vgf+/Vgf+ *mice, during day and night cycles, indicating that mechanism(s) other than hyperactivity were responsible for their increased energy expenditure. In *Vgf-/Vgf- *knockout mice, morphological analysis of brown and white adipose tissues (BAT and WAT) indicated decreased fat storage in both tissues, and decreased adipocyte perimeter and area in WAT. Changes in gene expression measured by real-time RT-PCR were consistent with increased fatty acid oxidation and uptake in BAT, and increased lipolysis, decreased lipogenesis, and brown adipocyte differentiation in WAT, suggesting that increased sympathetic nervous system activity in *Vgf-/Vgf- *mice may be associated with or responsible for alterations in energy expenditure and fat storage. In addition, uncoupling protein 1 (UCP1) and UCP2 protein levels, mitochondrial number, and mitochondrial cristae density were upregulated in *Vgf-/Vgf- *BAT. Using immunohistochemical and histochemical techniques, we detected VGF in nerve fibers innervating BAT and *Vgf *promoter-driven reporter expression in cervical and thoracic spinal ganglia that project to and innervate the chest wall and tissues including BAT. Moreover, VGF peptide levels were quantified by radioimmunoassay in BAT, and were found to be down-regulated by a high fat diet. Lastly, despite being hypermetabolic, VGF knockout mice were cold intolerant.

**Conclusion:**

We propose that VGF and/or VGF-derived peptides modulate sympathetic outflow pathways to regulate fat storage and energy expenditure.

## Background

The sympathetic nervous system (SNS) plays an important role in the regulation of glucose and fat metabolism, and its dysfunction could predispose to obesity and type 2 diabetes mellitus. Mice with defective signaling in the melanocortin pathway, including those that fail to express the hypothalamic melanocortin 4 receptor (MC4R) or its agonist alpha-melanocyte stimulating hormone (α-MSH), and those that over-express the MC4R antagonists agouti or agouti-related protein (AGRP or ART), all develop late onset obesity and insulin resistance [1]. The MC4R is expressed in the hypothalamic paraventricular nucleus (PVN), which contains neurons that innervate parasympathetic and sympathetic preganglionic cells in the brainstem and spinal cord, and throughout the brain in sympathetic circuits that innervate brown adipose tissue (BAT) [2,3]. These projections give the hypothalamus direct input to the autonomic nervous system, controlling glucose and energy homeostasis [4,5]. The central melanocortin system has therefore been proposed to modulate autonomic outflow and food intake [6,7] through its effects on sympathetic activity to inhibit pancreatic insulin secretion and increase energy utilization [8,9].

Brown and white adipose tissues (BAT and WAT) are sites where humoral factors (e.g. leptin and adiponectin) are synthesized and afferent nerve signals generated, which together convey the state of energy storage to the hypothalamus. These signals contribute to the adaptive responses that occur following changes in the environment, including diet and cold. BAT is the primary site of thermogenesis in rodents and newborn humans, is richly innervated by the SNS, and adipocytes are densely packed with mitochondria that contribute to energy generation and heat dissipation. The thermogenic capacity of BAT is primarily due to uncoupling protein 1 (UCP1) on the inner mitochondrial membrane that uncouples the respiratory chain from ATP generation, producing heat [10]. Norepineprine (NE), released from sympathetic nerve endings, stimulates lipolysis in WAT and increases UCP1 gene expression in BAT; these responses to a high fat diet or cold exposure are controlled at least in part by the central melanocortin system [3,11]. Increased UCP1 expression in WAT, the result of SNS stimulation or genetic background, is associated with brown adipocyte induction in WAT depots, and leads to reversal of diet-induced and genetic obesity [12-16]. Taken together, these data argue for an important role of the SNS in the control of energy expenditure via outflow to both white and brown adipose tissues.

We have previously reported suppression of obesity, hyperinsulinemia and hyperglycemia in A^y^/a agouti and melanocortin 4 receptor deficient (*MC4R-/MC4R-*) mice by targeted deletion of the *Vgf *gene [17,18]. VGF is a secreted protein and peptide precursor that is expressed throughout the brain, in neurons, and in several neuroendocrine and endocrine tissues [19,20]. Mice with a targeted deletion of *Vgf *are lean and hypermetabolic, and resist developing obesity and diabetes [21]. Neonatal treatment of *Vgf-/Vgf- *mice with monosodium glutamate, which damages the hypothalamus and the hypothalamic projections to the autonomic nervous system [22-24], blocks the development of the lean phenotype [17,18]. This suggests that VGF could regulate energy balance by reducing sympathetic outflow pathway activity to peripheral tissues. To gain insight into the molecular and cellular changes that may be responsible for the increase in energy expenditure and decrease in adipose stores detected in VGF knockout mice, we examined the effect that targeted ablation of VGF has on the regulation of gene expression in adipose tissues, analyzing two independent VGF knockout mouse lines on C57Bl6 backgrounds. We noted alterations in the expression of genes encoding enzymes that control fat synthesis and breakdown, consistent with increased SNS activity. We further found that VGF was expressed in neural pathways innervating BAT, quantified VGF-derived peptides in BAT by RIA, and demonstrated increased UCP1 and UCP2 levels in VGF knockout BAT that were associated with alterations in mitochondrial number and morphology. Our studies support the hypothesis that VGF modulates sympathetic outflow pathway activity to control fat storage and energy expenditure.

## Results

### Metabolic rates are higher, fat and lean body mass lower, and activity levels similar in *Vgf*-/*Vgf*- compared to *Vgf*+/*Vgf*+ mice on a C57Bl6 background

**Figure 1:**
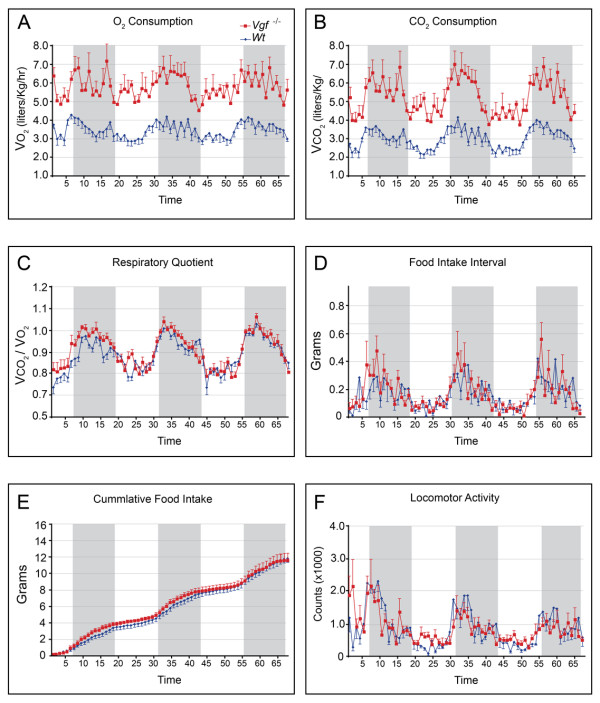
**VGF-deficient mice on a C57Bl6 background are hypermetabolic, with normal food intake and locomotor activity**. Metabolic cage analysis revealed that *Vgf*_*R *_*-/- *mice have significantly increased O_2 _(V0_2_, panel A) and CO_2 _(VCO_2_, panel B) consumption (two-tailed *t*-test; p ≤ 0.05), while respiratory quotient (panel C), food intake (panels D and E) and locomotor activity (panel F) were similar to wild type littermates (n = 7-8 mice of each genotype). Alternating white and grey shaded regions in panels E-F represent light rest and dark active periods, respectively.

**Figure 2:**
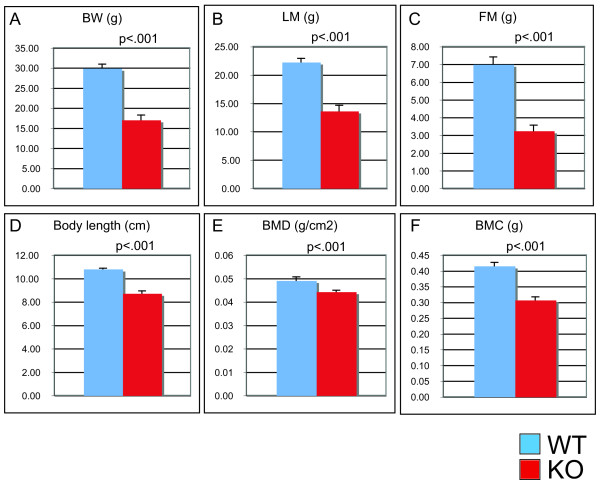
**VGF-deficient mice on a C57Bl6 background have decreased fat and lean body mass, and decreased bone mineral density and content**. Body weight (Panel A) (grams), body composition (Panels B and C) [lean mass (LM) and fat mass (FM)] (grams), length (Panel D) (cm), bone mineral density (BMD) (Panel E) (g/cm^2^) and bone mineral content (BMC) (Panel F) (grams), were determined in VGF_R _mice using dual emission x-ray absorption. Data are expressed as mean ± SEM (n = 7-8 male mice of each genotype per group).

Previous studies of VGF knockout mice on a mixed C57Bl6-129/SvJ background (designated VGF_Mixed _knockout and abbreviated VGF_M _knockout or *Vgf*_*M *_*-/-*) indicated that mice with the *Vgf *gene ablated were hyperactive and hypermetabolic. To investigate whether background strain had an effect on metabolic phenotype, we analyzed the same line of VGF knockout mice that had been backcrossed ≥ 10 generations to obtain a homogeneous C57Bl6 background (designated VGF_C57 _knockout or *Vgf*_*C*57 _*-/-*), as well as the F3 generation of an independent mouse line, generated by Regeneron Pharmaceuticals Inc. using F1H4 ES cells (a 129B6/F1-derived cell line), also primarily on a C57Bl6 background (>83% C57Bl6 background; designated VGF_R _knockout or *Vgf*_*R *_*-/-*) [25]. Similar results were obtained using both of these VGF mutant mouse lines on C57Bl6 backgrounds. As shown in Figure 1, metabolic rates remained high in homozygous VGF_R _knockout mice, with basal metabolic rate (BMR), defined as oxygen consumption (V0_2_) during the light rest period, significantly elevated (p < 0.001; two tailed *t*-test) in VGF null compared to wild-type littermates. Locomotor activity levels and food intake were indistinguishable between control mice and either VGF_R _(Figure 1) or VGF_C57 _(data not shown) knockout mice. Body weight and length were reduced in VGF_R _and VGF_C57 _knockout mice [Figure 2 and [18], respectively], and body mass analysis indicated a reduction in fat and lean mass (Figure 2A-C). Consistent with reduced fat and lean mass, low leptin levels, and the hypothesized increase in sympathetic tone in VGF mutant mice, significant reductions in both bone density and bone mineralization were also noted in VGF_R _knockout mice (Figure 2E-F). Serum chemistries revealed significant reductions in HDL, triglyceride, albumin, and leptin in VGF_R _knockout mice, while total cholesterol, total protein, insulin, LDL, creatinine, magnesium, urea, and uric acid were not significantly different between knockout and wild type mice on the C57Bl6 background (Table 1).

**Table 1:** Serum measures in wild type (*Vgf*_*R *_+/+) and VGF knockout (*Vgf*_*R *_-/-) mice.

	***Vgf*_*R *_+/+**	***Vgf*_*R *_-/-**	***P *value**
Insulin (ng/mL)	0.82 ± 0.14	0.67 ± 0.19	0.55
Leptin (pg/mL)	2351 ± 546	399 ± 80	0.003*
Albumin (g/dL)	3.02 ± 0.07	2.74 ± 0.04	0.01*
Total Protein (g/dL)	5.24 ± 0.12	4.98 ± 0.09	0.10
Creatinine (mg/dL)	0.43 ± 0.02	0.39 ± 0.01	0.09
Magnesium (mg/dL)	2.43 ± 0.08	2.33 ± 0.07	0.35
Triglyceride (mg/dL)	80.29 ± 9.31	46.03 ± 3.24	0.003*
Urea (mg/dL)	20.94 ± 0.99	20.45 ± 0.62	0.68
Uric Acid (mg/dL)	1.41 ± 0.14	1.50 ± 0.17	0.69
Cholesterol (mg/dL)	90.25 ± 5.86	76.18 ± 3.37	0.06
LDL (mg/dL)	4.36 ± 0.57	5.21 ± 0.57	0.31
HDL (mg/dL)	53.33 ± 4.80	40.20 ± 2.05	0.02*

Chemistry analyses were carried out on serum samples as previously described [103]. Data are expressed as mean ± SEM; n = 8 for each genotype. *P *values of less than 0.05 were considered significant and are denoted by the *.

### Morphological changes in BAT and WAT of homozygous VGF mutant mice suggest an increase in lipolysis and FA oxidation

**Figure 3:**
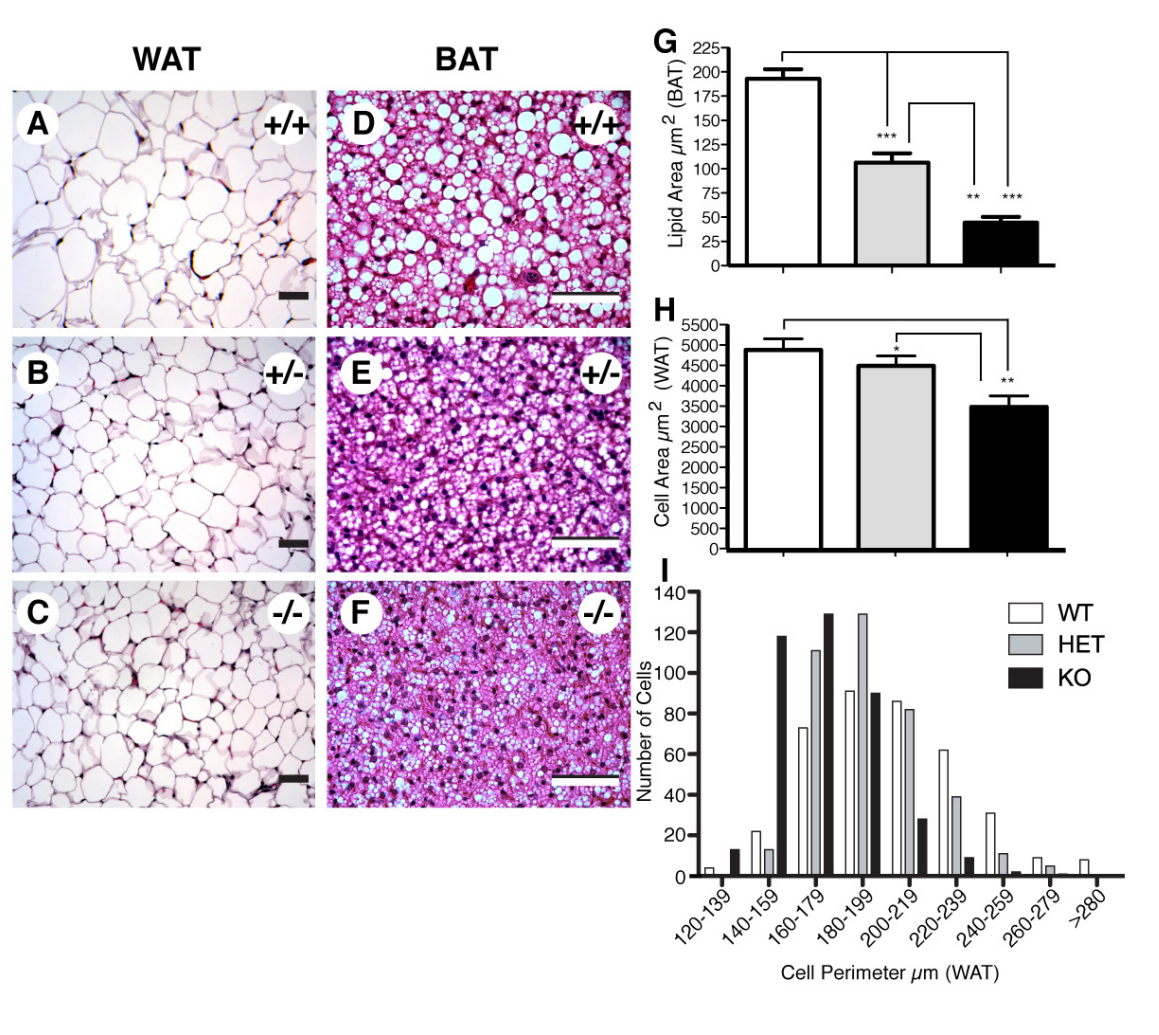
**Lipid deposition in multilocular cells of brown adipose tissue (BAT) and unilocular cells of white adipose tissue (WAT) is reduced in VGF knockout mice**. Representative sections of epididymal WAT (A-C) and interscapular BAT (D-F) from ad lib-fed, age-matched *Vgf*_*R *_*+/+ *(A, D), +/- (B, E) and *-/- *(C, F) were stained with H&E. Adipocytes in interscapular BAT and epididymal WAT from *-/- *mice (C and F) contain less lipid accumulation than the corresponding adipocytes from wild type mice (A and D). Scale bars are 50 μm in length. Morphometric analysis of adipose tissue from *Vgf*_*R *_*+/+*, +/-, and *-/- *mice was carried out using MetaMorph, as detailed in Materials and Methods. Lipid area in BAT (panel G), cell area in WAT (panel H), and the distribution of adipocyte perimeters in WAT (panel I), were quantified for each genotype. Note the significant reduction in lipid area in both +/- and *-/- *BAT (ANOVA, F (2,15) = 73.5, p < 0.0001), significant reduction in adipocyte area in *-/- *WAT (ANOVA, F (2,15) = 7.6, p = 0.005), and shift in the distribution of cells favoring those with significantly smaller perimeters in *-/- *and +/- WAT (comparison of mean perimeters in μm; ANOVA, F (2,1167) = 166, p < 0.0001), compared to wild type. Values are mean ± SEM, *p < 0.05, **p = 0.005, ***p < 0.0001 compared to wild type (ANOVA and Bonferroni's posthoc test).

Histological examinations of adipose tissues in *Vgf -/- *and wild type mice were performed to gain insight into which tissues may be responsible for increased energy expenditure and/or decreased fat stores. We compared tissues from *Vgf*_*R *_*-/-*, *-/+*, and *+/+ *mice, although previous studies have not demonstrated significant metabolic differences between wild type and heterozygous VGF knockout mice [17,18,21]. Because fat mass is significantly reduced in *Vgf*_*C*57 _and *Vgf*_*R *_*-/- *mice (e.g. Figure 2), certain fat depots were extremely difficult to identify and quantify on the C57Bl6 background, including visceral mesenteric fat, subcutaneous abdominal fat, and retroperitoneal fat. Even though these various fat depots are differentially responsive to a number of stimuli (e.g. diet), and are thought to make specific contributions to glucose and metabolic homeostasis [26], we chose to focus on epididymal WAT and interscapular BAT pads for histological and gene expression analyses because they could be reproducibly isolated from knockout mice. BAT and WAT have distinct physiological roles in maintaining energy homeostasis; BAT is the most highly metabolic and innervated tissue in rodents, while WAT is the principal site of adipose storage. The BAT of *Vgf*_*C*57 _*-/-*, *Vgf*_*R *_*-/-*, *Vgf*_*R *_*+/-*, and wild type mice was comprised of typical multilocular cells, however, these cells in VGF knockout mice contained large numbers of smaller lipid droplets, suggesting that the tissue remained metabolically active (Figure 3D-F; quantification panel G; Additional file 1: Supplemental Figure 1). Moreover, the WAT depot that normally surrounds BAT in wild type mice was absent in *Vgf -/- *mice (see Additional files 1 and 2: Supplemental Figures 1 and 2). WAT from *Vgf -/- *mice contained less stored lipid in comparison to wild type mice (Figure 3, C and 3A), consistent with the body composition fat mass analysis (Figure 2). Morphometric analysis indicated that mean adipocyte area was significantly reduced in knockout WAT (Figure 3H), which was associated with a shift in the distribution of adipocytes favoring those with smaller perimeters (Figure 3I). In general, quantitative analysis of WAT and BAT demonstrated that heterozygous VGF_R _knockout mice had an intermediate phenotype between wild type and homozygous knockout mice (Figure 3G and 3H). Overall, H&E analysis of *Vgf*_*C*57 _*-/- *BAT and WAT (Additional file 1: Supplemental Figure 1) revealed a similar pattern of histological staining to that obtained with the respective *Vgf*_*R *_*-/- *tissues (Figure 3).

### *Vgf*-/*Vgf*- mice have altered adipose mRNA levels encoding proteins involved in the regulation of energy storage, fatty acid utilization, and sympathetic nervous system activity

The adipose tissues of VGF_C57 _and VGF_R _mutant mice were morphologically different from wild type mice. To understand the molecular mechanisms responsible for decreased fat stores in epididymal WAT and interscapular BAT, we carried out an extensive analysis of gene expression in these VGF_C57 _tissues using real-time RT-PCR. In WAT, the levels of mRNAs, encoding enzymes responsible for building fat stores, acetyl coA carboxylase (ACC), glycerol-3-phosphate acyltransferse (GPAT) and diacylglycerol acyltransferase (DGAT), were unaltered in *Vgf -/- *mice, while an increase in mRNA levels encoding hormone sensitive lipase (HSL), an enzyme responsible for lipolysis which mobilizes fat, was found in *Vgf -/- *mice. Gene expression of peroxisome-proliferator activated receptor gamma (PPARγ), a protein that promotes adipocyte differentiation, and sterol regulatory element binding protein 1C (SREBP-C1), which activates genes responsible for lipogenesis and glucose utilization [27], were decreased in VGF knockout WAT. RNA levels encoding fatty acid synthase (FAS), an enzyme that synthesizes fatty acids from acetyl COA and malonyl COA, however, was elevated in *Vgf -/- *WAT. In VGF knockout BAT, levels of mRNAs encoding the lipogenic proteins ACC, GPAT and PPARγ were increased (Table 2).

**Table 2:** Expression of genes in epididymal WAT and interscapular BAT that contribute to lipolysis or lipogenesis in VGF knockout and wild type mice.

**Tissue**	**Gene**	**Mean Cycle Threshold (C_t_) Value**	**Std. Error**	**Fold Difference in mRNA Levels (2^ΔΔCt^)**	**P-Value**	**RNA Levels in VGF_C57_** **-/- vs +/+ mice**
BAT	ACC	*Vgf-/Vgf- *2.57 *Vgf+/Vgf+ *1.52	.596 .205	2.1	NS	

BAT	DGAT	*Vgf-/Vgf- *3.45 *Vgf+/Vgf+ *3.52	.210 .092	1.0	NS	

BAT	GPAT	*Vgf-/Vgf- *6.95 *Vgf+/Vgf+ *8.12	.253 .127	2.3	.0060	Increased

BAT	PPARγ	*Vgf-/Vgf *-.633 *Vgf+/Vgf+ *1.73	.285 .239	2.1	.002	Increased

WAT	ACC	*Vgf-/Vgf- *5.20 *Vgf+/Vgf+ *3.39	.517 .333	3.5	.001	Decreased

WAT	DGAT	*Vgf-/Vgf- *6.88 *Vgf+/Vgf+ *7.88	.370 .525	2.0	NS	

WAT	GPAT	*Vgf-/Vgf- *7.83 *Vgf+/Vgf+ *9.01	.458 1.13	2.2	NS	

WAT	PPARγ	*Vgf-/Vgf- *4.75 *Vgf+/Vgf+ *3.60	.315 .138	2.2	.010	Decreased

WAT	FAS	*Vgf-/Vgf- *2.45 *Vgf+/Vgf+ *4.80	.193 .227	5.1	.0098	Increased

WAT	SREBP1C	*Vgf-/Vgf- *12.6 *Vgf+/Vgf+ *8.99	.953 .560	12.1	.001	Decreased

WAT	HSL	*Vgf-/Vgf- *7.79 *Vgf+/Vgf+ *11.6	.405 1.10	14.0	.022	Increased

Real time RT-PCR analysis was carried out in quadruplicate as described in the Methods. The fold difference was calculated from the indicated mean cycle threshold (C_t_) values, the amplification cycle when product can first be detected: lower C_t _values correspond to greater abundance. C_t _values were normalized to the expression of the housekeeping gene beta-actin (ΔΔCt), and fold difference in mRNA calculated as 2^ΔΔCt^, as detailed in Methods, with p ≤ 0.05 considered significant.

Next we looked at gene products responsible for fatty acid beta-oxidation in these tissues. RNA levels of proteins responsible for fatty acid and glucose uptake, lipoprotein lipase (LPL) and glucose 4 transporter (GLUT 4), and translocation of fatty acids to the mitochondria, carnitine palmitoyltransferase 1 (CPT1), were up regulated in *Vgf -/- *BAT, whereas LPL and GLUT4 gene expression were down regulated and CPT1 mRNA levels unaltered in WAT. PPARα is thought to play an important role in lipid metabolism, and mRNA levels were increased in VGF knockout BAT and WAT. Furthermore, mRNA levels encoding PPARγ coactivator 1 (PGC-1), a co-activator of nuclear receptors that increases metabolism by stimulating mitochondrial biogenesis and respiration, were up-regulated in BAT and WAT of the *Vgf*_*C*57 _*-/- *mutant mouse, and a concomitant increase in cytochrome C oxidase II (COX II) mRNA levels was also found (Table 3).

**Table 3:** Expression of genes in epididymal WAT and interscapular BAT that contribute to fatty acid oxidation in VGF knockout and wild type mice.

**Tissue**	**Gene**	**Mean Cycle Threshold (C_t_) Value**	**Std**. **Error**	**Fold Difference in mRNA Levels (2^ΔΔCt^)**	**P-Value**	**RNA Levels in VGF_C57_** **-/- vs +/+ mice**
BAT	PPARα	*Vgf-/Vgf- *5.23 *Vgf+/Vgf+ *5.83	.433 .222	1.5	NS	

BAT	LPL	*Vgf-/Vgf- *2.33 *Vgf+/Vgf+ *3.48	.259 .263	2.2	.021	Increased

BAT	CPT-1	*Vgf-/Vgf- *13.9 *Vgf+/Vgf+ *15.3	.546 .312	2.6	.05	Increased

WAT	PGC-1	*Vgf-/Vgf- *10.1 *Vgf+/Vgf+ *12.2	.431 .271	4.3	.0028	Increased

WAT	COX II	*Vgf-/Vgf- *1.91 *Vgf+/Vgf+ *2.67	.207 .048	1.7	.019	Increased

WAT	PPARα	*Vgf-/Vgf- *11.9 *Vgf+/Vgf+ *14.3	.494. .112	5.3	.0069	Increased

WAT	LPL	*Vgf-/Vgf- *4.09 *Vgf+/Vgf+ *0.96	1.13 .111	2.0	.0232	Decreased

WAT	CPT-1	*Vgf-/Vgf- *16.3 *Vgf+/Vgf+ *16.7	.543 .427	1.3	NS	

Real time RT-PCR analysis was carried out in quadruplicate as described in the Methods. The fold difference was calculated from the indicated mean cycle threshold (C_t_) values, the amplification cycle when product can first be detected: lower C_t _values correspond to greater abundance. C_t _values were normalized to the expression of the housekeeping gene beta-actin (ΔΔCt), and fold difference in mRNA calculated as 2^ΔΔCt^, as detailed in Methods, with p ≤ 0.05 considered significant.

We then investigated whether altered BAT morphology could be associated with an increase in thermogenesis by examining mRNA levels for the uncoupling proteins. Uncoupling Protein 1 (UCP1) by disrupting the proton gradient across the inner mitochondrial membrane is crucial for non-shivering thermogenesis, leading to heat production (reviewed in [28-30]). UCP2 and UCP3 also regulate proton conductance, however they require activation by reactive oxygen species, and may protect against oxidative damage (reviewed in [28-30]). In addition, UCP3 may transport fatty acids, while UCP2 may attenuate insulin secretion in pancreatic beta cells. In VGF_C57 _knockout mice compared to wild type mice, we noted that mRNA levels of UCP1 were lower in BAT, but the mRNA levels of UCP2 and UCP3 were increased (Table 4). Notably, in WAT, levels of UCP1 mRNA were robustly increased in *Vgf -/- *mice (Table 4).

**Table 4:** Expression of genes in epididymal WAT and interscapular BAT that contribute to thermogenesis in VGF knockout and wild type mice.

**Tissue**	**Gene**	**Mean Cycle Threshold (C_t_) Value**	**Std**. **Error**	**Fold Difference in mRNA Levels (2^ΔΔCt^)**	**P-Value**	**RNA Levels in VGF_C57_** **-/- vs +/+ mice**
BAT	UCP1	*Vgf-/Vgf- *3.53 *Vgf+/Vgf+ *1.22	.843 .272	4.9	.024	Decreased

BAT	UCP2	*Vgf-/Vgf- *1.88 *Vgf+/Vgf+ *4.15	.259 .233	4.8	.0013	Increased

WAT	UCP1	*Vgf-/Vgf- *11.2 *Vgf+/Vgf+ *14.4	.433 .527	9.2	.0031	Increased

WAT	UCP2	*Vgf-/Vgf- *3.02 *Vgf+/Vgf+ *3.86	.535 .275	1.8	.025	Increased

Real time RT-PCR analysis was carried out in quadruplicate as described in the Methods. The fold difference was calculated from the indicated mean cycle threshold (C_t_) values, the amplification cycle when product can first be detected: lower C_t _values correspond to greater abundance. C_t _values were normalized to the expression of the housekeeping gene beta-actin (ΔΔCt), and fold difference in mRNA calculated as 2^ΔΔCt^, as detailed in Methods, with p ≤ 0.05 considered significant.

Lastly, we looked at genes indicative of sympathetic nervous system (SNS) activity. The SNS-mediated physiological responses, FA oxidation and lipolysis, mostly rely on release of norepinephrine from the post-ganglionic neurons which binds to beta-3-adrenergic receptors (β3AR) on the adipocyte's cell surface, as well as to the other beta receptors, β1AR and β2AR. Mice with decreased sympathetic activity due to leptin deficiency have a ~300-fold reduction in β3AR mRNA in comparison to lean animals [31,32]. Therefore we looked at the mRNA levels of the beta-adrenergic receptors to provide indirect support for an increase in SNS tone in VGF mutant mice. In BAT, β3AR mRNAs were increased in VGF knockout mice in comparison to wild type mice. In WAT, however, the gene expression of β2AR was significantly higher in *Vgf -/- *mice than *Vgf +/+ *mice, while β3AR mRNA levels were similar (Table 5).

**Table 5:** Expression of genes in epididymal WAT and interscapular BAT that contribute to sympathetic nervous system activity in VGF knockout and wild type mice.

**Tissue**	**Gene**	**Mean Cycle Threshold (C_t_) Value**	**Std**. **Error**	**Fold Difference in mRNA Levels (2^ΔΔCt^)**	**P-Value**	**RNA Levels in VGF_C57_** **-/- vs +/+ mice**
BAT	β3AR	*Vgf-/Vgf- *2.55 *Vgf+/Vgf+ *2.90	.456 .461	1.3	NS	

BAT	β2AR	*Vgf-/Vgf- *4.50 *Vgf+/Vgf+ *7.54	.730 .204	8.0	.0029	Increased

BAT	β1AR	*Vgf-/Vgf- *8.41 *Vgf+/Vgf+ *8.13	.222 .193	1.2	NS	

WAT	β3AR	*Vgf-/Vgf- *2.35 *Vgf+/Vgf+ *1.37	.750 .278	1.9	NS	

WAT	β2AR	*Vgf-/Vgf- *5.71 *Vgf+/Vgf+ *7.21	.361 .192	2.8	.0104	Increased

WAT	β1AR	*Vgf-/Vgf- *4.43 *Vgf+/Vgf+ *5.45	.634 .143	2.0	NS	

Real time RT-PCR analysis was carried out in quadruplicate as described in the Methods. The fold difference was calculated from the indicated mean cycle threshold (C_t_) values, the amplification cycle when product can first be detected: lower C_t _values correspond to greater abundance. C_t _values were normalized to the expression of the housekeeping gene beta-actin (ΔΔCt), and fold difference in mRNA calculated as 2^ΔΔCt^, as detailed in Methods, with p ≤ 0.05 considered significant.

### Western and electron microscopic analyses indicate that UCP1 and UCP2 protein levels and mitochondrial number are upregulated in VGF knockout BAT

**Figure 4:**
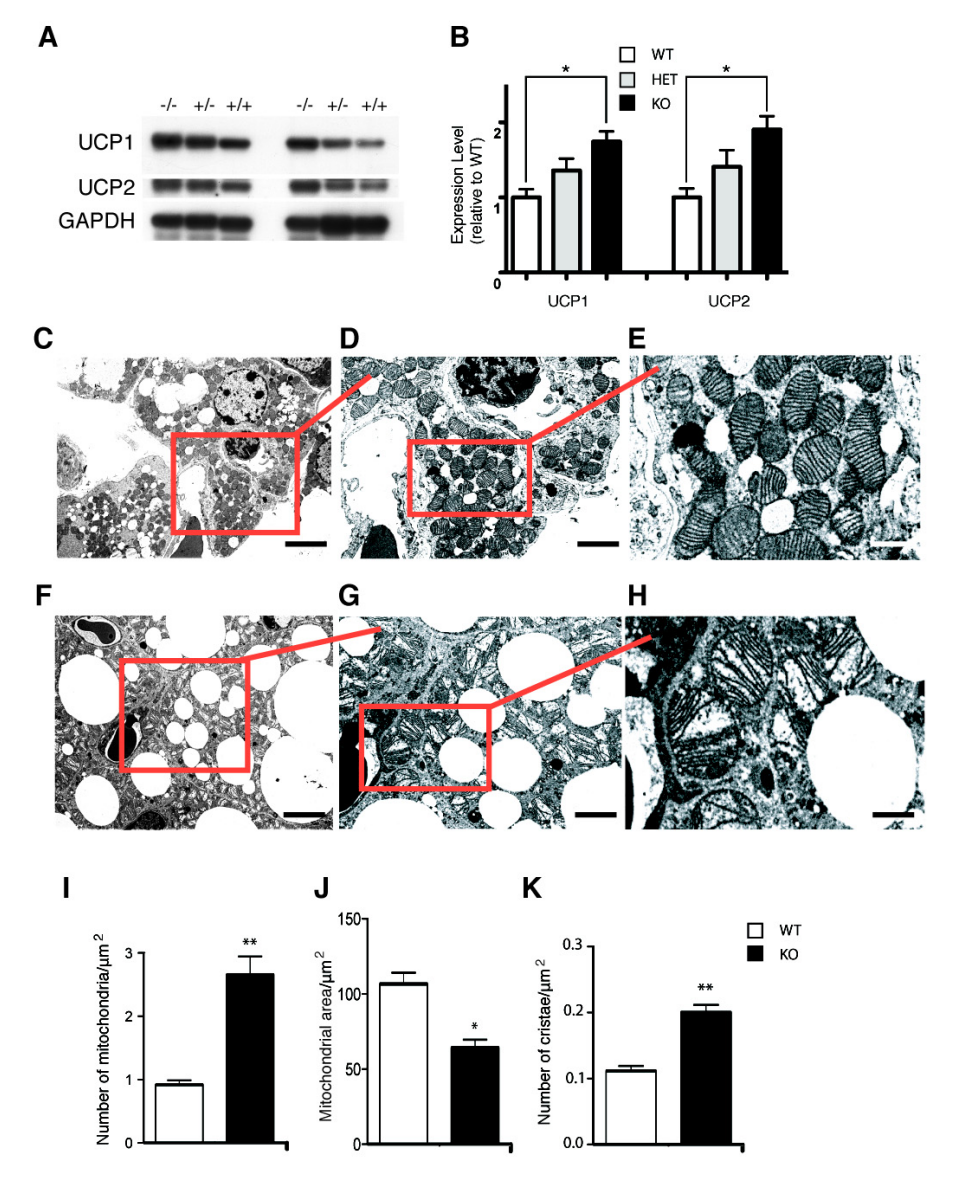
**VGF ablation affects UCP1 & UCP2 protein expression, mitochondrial morphology, and mitochondrial number in BAT**. Representative western blots of UCP1, UCP2, and GAPDH protein levels in BAT from *Vgf*_*R *_*+/+*, +/-, and *-/- *mice (panel A), and densitometric quantification by NIH image (panel B). Comparable results were found in at least three additional experiments. Ablation of VGF significantly increased UCP1 and UCP2 levels in BAT from *Vgf*_*R *_-/- but not *Vgf*_*R *_+/- mice compared to wild type +/+ mice [n = 5 mice per group, mean ± SEM, F (2,12) = 4.35 (UCP1), F (2,12) = 3.33 (UCP2), *p < 0.05, ANOVA and Dunnett's posthoc test]. Representative electron microscope photomicrographs of BAT from *Vgf*_*R *_-/- (panels C-E) and +/+ mice (panels F-H) are shown. The part of the image that is boxed in red is shown at higher magnification in the panel to its immediate right. Scale bars are 5 μm (panels C and F), 2.5 μm (panels D and G), and 1.25 μm (panels E and H). Note that mitochondria are smaller and more numerous in *Vgf*_*R *_-/- in comparison to +/+ BAT (compare panels E and H), which is quantified in panels I and J. In panel K, the number of cristae per μm^2 ^of mitochondrial area was quantified in *Vgf*_*R *_-/- and +/+ BAT. Values are mean ± SEM; ***p = 0.0002 (panel E), **p = 0.001 (panel F), ***p < 0.0001 (panel G) (Student's t-test).

To determine the basis of the hypermetabolic state in VGF knockout mice, given the somewhat surprising lack of an increase in UCP1 mRNA levels in BAT (Table 4), we examined protein levels of UCPs in BAT by western analysis, and quantified mitochondrial size and number, and cristae density, by electron microscopy. UCP1 and UCP2 protein levels were significantly increased in BAT from *Vgf*_*R *_*-/- *knockout compared to wild type mice (Figure 4, panels A and B; p < 0.05, ANOVA). To determine whether mitochondrial biogenesis could be affected by germline *Vgf *ablation, knockout and wild type BAT was examined by electron microscopy (Figure 4, panels C-E and F-H, respectively). Mitochondrial number was increased (Figure 4I), mitochondrial area decreased (Figure 4J), and cristae density increased (Figure 4K), in VGF -/- knockout compared to +/+ wild type mice.

### VGF immunoreactivity can be detected in BAT and VGF-derived AQEE30 peptide levels are regulated by diet

**Figure 5:**
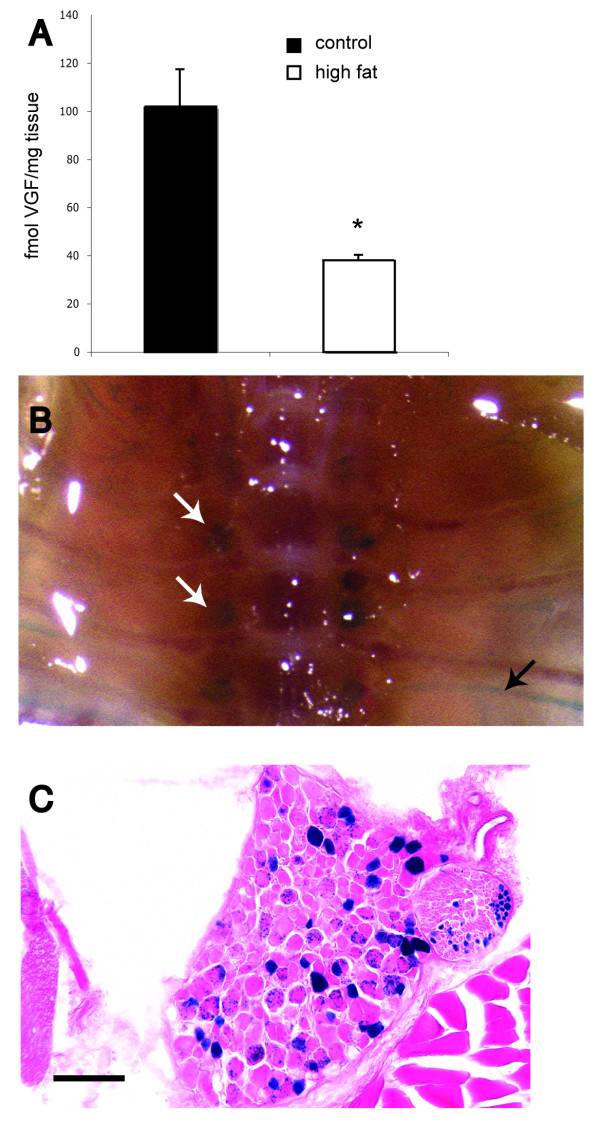
**VGF is expressed in neural pathways that innervate brown adipose tissue (BAT)**. In panel A, levels of VGF-derived peptides were quantified in BAT from C57Bl6 female mice fed standard chow or a high fat, high carbohydrate diet for 14 days. BAT was removed and RIA analysis carried out to quantify C-terminal VGF-derived peptides (NAPP129, TLQP62 and AQEE30, but not full length VGF), using polyclonal anti-AQEE30 (VGF_588-617_) antiserum as previously described [38]. VGF peptide levels in BAT were significantly reduced in mice fed the high-energy diet in comparison to control mice (p < 0.05). Comparisons by ANOVA [mean ± SEM; high fat diet (n = 4), standard diet (n = 3)]. In panels B-C, *Vgf *gene expression was visualized in neural pathways that innervate tissues including BAT using a *lacZ *reporter gene that was knocked into the mouse *Vgf *locus in VGF_R _mice (see Methods). In panel B, β-galactosidase expression (blue staining) in the dorsal root ganglia (white arrows) and intercostal nerve (black arrow) is seen in a whole mount preparation from a heterozygous *Vgf*_*R *_*+/- *mouse. A dorsal root ganglion section is shown at higher magnification in panel C (scale bar is 100 μm in length).

BAT is a highly metabolic tissue that is innervated by noradrenergic sympathetic fibers, which also contain other neuropeptides such as NPY [33-37]. As previous studies suggested that VGF ablation could potentially impact sympathetic nervous system activity to peripheral metabolic tissues such as fat, and for technical reasons immunohistochemical visualization of VGF in WAT was difficult, we investigated whether VGF was expressed in nerve fibers that innervate BAT. We first quantified the level of VGF-derived C-terminal peptides by subjecting acid extracts of BAT to a sensitive radioimmunoassay (RIA) that has been previously described [38]. This RIA utilizes a polyclonal antibody specific for the VGF-derived C-terminal peptide AQEE30 (VGF peptides are designated by the four N-terminal amino acids and length), so detects AQEE30 and the N-terminally extended peptides that contain this sequence, TLQP62 and NAPP129 [38]. Immunoreactive AQEE30 was detected in BAT, and was found to be downregulated when C57Bl6 mice were fed a high fat diet for two weeks (Figure 5A). Parenchymal nerve fibers make direct contact with adipocytes, activating them via beta-adrenergic receptors, or are associated with finely branched vessels that penetrate the tissue but do not make direct contacts with the adipocytes [39,40]. By immunofluorescence and immunohistochemistry, anti-VGF staining was found in BAT to abut adipocytes and was also seen in the periarterial plexus (Additional file 3: Supplemental Figure 3, panels A, C, D), similar to the distribution reported for anti-tyrosine hydroxylase (Additional file 3: Supplemental Figure 3, panel B), which binds the rate-limiting enzyme in catecholamine biosynthesis and is a marker of catecholaminergic nerves and terminals [37]. Consistent with finding VGF in nerve fibers and terminals that innervate BAT, we were also able to indirectly localize *Vgf *gene expression in spinal ganglia and intercostal nerves of heterozygous knockout mice. Intercostal nerves, which contain autonomic and sensory fibers that innervate BAT [41] and additional structures including the skin and musculature of the chest and abdomen, and the dorsal root ganglia, which give rise to the somatic and sensory afferents in the intercostal nerve, were found to express β-galactosidase from the *lacZ *reporter gene that was knocked into the mouse *Vgf *locus in *Vgf*+/*Vgf*-^*lacZ *^mice (Figure 5, panels B-C). These initial data suggest that VGF and/or VGF-derived peptides could be released from neurons that innervate adipose tissues, as is NPY [42-44], and that VGF AQEE30 peptide levels in BAT are regulated by diet; additional studies will be required to determine whether adipocytes synthesize VGF, analogous to recent reports that they produce NPY [45,46].

### VGF knockout mice are extremely sensitive to acute cold stress

**Figure 6:**
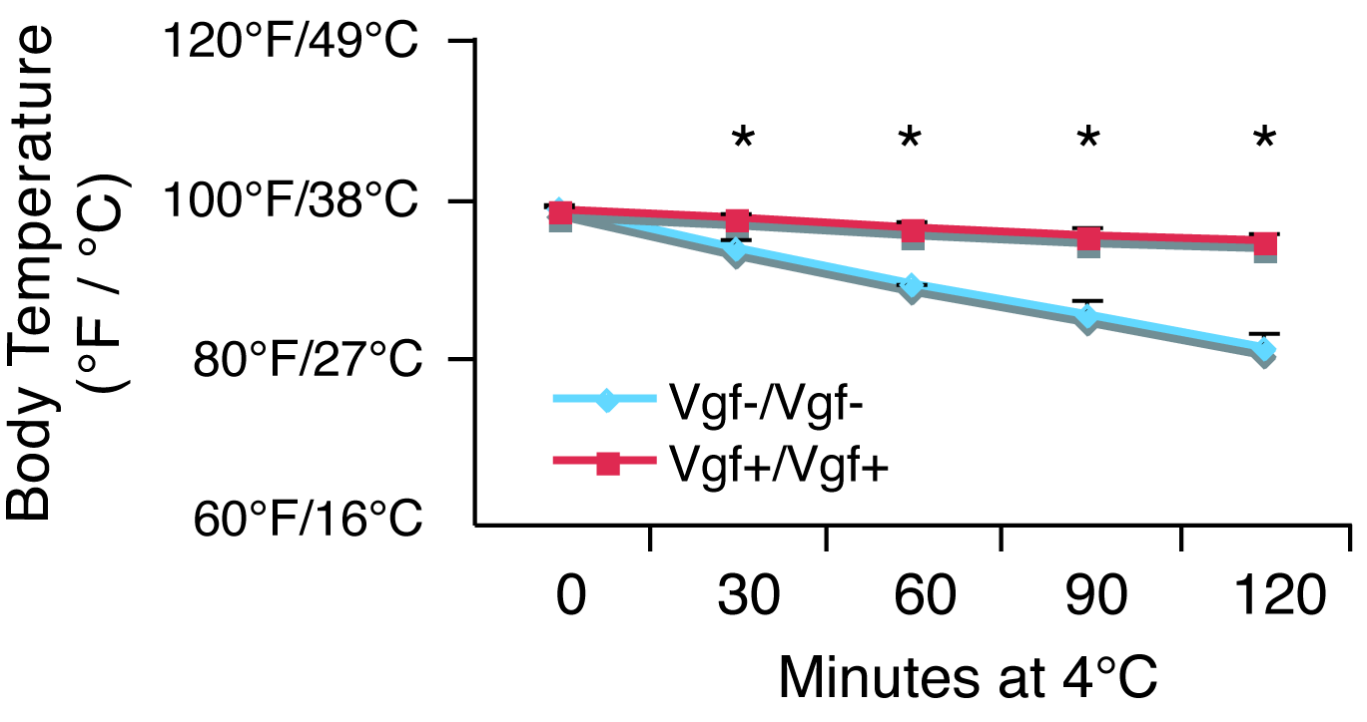
**VGF-deficient mice have increased sensitivity to cold exposure**. *Vgf*_*C*57 _*-/- *and *Vgf*_*C*57 _*+/+ *mice were placed at 4°C for 2 hours and allowed adlib access to food and water. Mice were individually housed and rectal temperatures were taken every 30 minutes. Body temperatures that are marked with an asterix from mutant (n = 2) and wild-type mice (n = 5) are significantly different from each other (ANOVA; p < 0.05; mean ± SEM).

In rodents, the principal functions of BAT are to generate heat during cold exposure so as to maintain body temperature (cold-induced thermogenesis), and to dissipate excess energy in response to dietary intake (diet-induced thermogenesis). To determine whether VGF ablation affected BAT function and thermogenesis, *Vgf*_*C*57 _*-/- *and *Vgf*_*C*57 _*+/+ *mice were acutely exposed to the cold. In contrast to wild type mice that were able to maintain body temperature, VGF knockout mice despite being hypermetabolic rapidly became hypothermic at 4°C (Figure 6).

## Discussion

In this study, we investigated the effect that targeted ablation of VGF has on peripheral adiposity, and the possible mechanism(s) by which this neuronal, endocrine, and neuroendocrine secreted polypeptide might regulate fat storage and autonomic homeostasis. VGF mutant mice on a C57Bl6 background are hypermetabolic and lean, with reduced lean and fat mass, despite normal food consumption and locomotor activity levels (Figures 1 and 2). These current findings are consistent with previous studies of VGF mutant mice on a mixed C57Bl6/sv129 background [21]. Thus, serum chemistries, insulin-tolerance, glucose-tolerance, metabolic measurements, and body composition are all very similar among VGF knockout mice on mixed (designated VGF_M_), homogeneous C57Bl6 (designated VGF_C57_), or ~83% C57Bl6 backgrounds (designated VGF_R_) [17,18] and this report], with the notable exception that VGF knockout mice on a C57Bl6 background (VGF_R _and VGF_C57_) are not hyperactive (Figure 1 and data not shown), and thus locomotor activity cannot play a substantive role in their increased energy expenditure.

Could increased sympathetic tone or sensitivity to norepinephrine be responsible for alterations in adipose morphology and the expression of critical gene products? Decreased lipid accumulation in WAT, smaller interscapular WAT depots that are associated with BAT, and regions of fat accretion in BAT, are consistent with increased sympathetic nervous system activity. In addition, changes in gene expression in WAT and BAT correlate with these morphological differences, as does body composition analysis. Activation of the sympathetic nervous system promotes catabolism by increasing glucose and fatty acid uptake into BAT [47], and by increasing lipolysis in WAT. Hormone sensitive lipase (HSL) is induced by sympathetic activity [48], is up-regulated by fasting in most adipose depots except subcutaneous fat [49-51], and both HSL mRNA and protein levels in adipose tissue are decreased in the obese insulin-resistant state [52]. Of note, HSL was one of the most robustly up-regulated genes in WAT from *Vgf*_C57 _*-/- *mice (Table 2). Although lipoprotein lipase (LPL) activity is generally regulated at the post-transcriptional level in adipose tissues (e.g. [53]), allowing rapid responses to alterations in diet, LPL mRNA levels correlate with PPARγ mRNA levels in epididymal WAT [54] and increase with PPARγ agonist treatment [55], likely due to a transcriptionally active PPAR response element upstream of the LPL gene [56]. Small but significant decreases in LPL mRNA and PPARγ mRNAs were noted in WAT from VGF knockout mice, while small increases in both mRNA levels were measured in BAT. Increased LPL expression in BAT has previously been described in mice exposed to cold or injected with norepinephrine [57], so the increased LPL expression we measured in VGF_C57 _knockout BAT may reflect increased sympathetic pathway activity. Decreased fat stores could result from continuous breakdown of triglycerides into fatty acids, which is associated with increased fatty acid synthase (FAS) mRNA levels in WAT and higher circulating FFA levels previously noted in *Vgf-/Vgf- *mice on a C57Bl6 background [18]. We further noted increases in cytochrome c oxidase II, PPARα, PGC1, UCP1, and UCP2 mRNA levels in WAT from VGF knockout mice. The PGC1 coactivators play essential roles in BAT differentiation, mitochondrial biogenesis, and thermogenesis [58], so increased expression of PGC1 could drive alterations in WAT morphology and UCP1 gene expression towards a 'brown fat' phenotype in *Vgf*_*C*57 _*-/- *mice.

Previous studies indicate that induction of UCP1 levels in the absence of increased adrenergic activity does not necessarily lead to increased UCP1 activity and thermogenesis [59,60]. Conversely, the thermogenic capacity of BAT can be augmented without an associated increase in sympathetic activity, as noted in the Syrian hamster exposed to a short photoperiod or fed a high-energy diet, where increased thermogenesis occurs in the absence of changes in norepinephrine turnover [61-63]. Our metabolic studies of VGF mutant mice consistently demonstrate increased O_2 _consumption irrespective of genetic background ([21] and this report), and together with the current analysis of gene expression, would be most consistent with increased fatty acid oxidation and decreased lipogenesis in adipose tissues, and possibly also thermogenic activity due to enhanced UCP1 levels in WAT, which could be associated with increased adrenergic activity. In BAT, targeted ablation of VGF resulted in increased UCP1 protein levels, increased mitochondrial number, and increased cristae number per square micron of mitochondrial area, suggesting increased sympathetic activity, but did not increase cold tolerance. Cold intolerance (Figure 6) in VGF mutants could be a result of lack of available fuel, as in the adlib fed state, these mice have reduced fat depots and display a fasted phenotype, with little detectable liver glycogen, low circulating glucose levels, increased circulating FFA, elevated hypothalamic NPY and AGRP mRNA levels, and decreased hypothalamic POMC mRNA levels [17,18,21]. Alternatively, sympathetic outflow to various adipose depots could differ, as has been demonstrated for specific WAT pads during prolonged fasting and following leptin administration [64,65].

Neonatal monosodium glutamate (MSG) blocks the development of the lean phenotype in VGF_M _knockout mice [17], suggesting that increased SNS activity is associated with increased fat oxidation and energy expenditure in homozygous *Vgf-/Vgf- *mice. Neonatal guanethidine treatment, which creates a generalized chemical sympathectomy, also blocked the development of the lean phenotype in VGF_C57 _knockout mice, and increased circulating glucose levels (see Additional file 2: Supplemental Figure 2), but was not sufficiently well-tolerated by VGF knockout mice to carry out a complete analysis of its effects. These experiments are consistent with the real-time PCR data, and a role for increased sympathetic pathway activity in mice that lack VGF. Although we were unable to study the developing SNS in *Vgf-/Vgf- *P0 newborn pups due to insufficient numbers, we did note that tyrosine hydroxylase (TH) immunostaining in the SNS of *Vgf*_*C*57 _*+/- *pups was significantly more robust than *Vgf*_*C*57 _*+/+ *pups, consistent with a possible increase in SNS catecholamine turnover (E. Watson and S. Salton, unpublished data) [66]. Increased sympathetic tone in the absence of changes in BAT UCP1 gene expression was noted in lean, hypermetabolic M_3 _muscarinic acetylcholine receptor knockout mice [67], which are very similar to VGF mutant mice in phenotype, and also largely resistant to high fat diet-induced, gold-thio-glucose-induced and genetically (*ob/ob*)-induced obesity, hyperphagia, hyperinsulinemia, and hyperglycemia [17,18,21,67].

Both cold exposure and overeating increase sympathetic drive to BAT to stimulate thermogenesis [68-70]. Are the sympathetic pathways that control increased energy expenditure and decreased fat storage regulated by independent CNS circuits? Adipose-synthesized leptin controls energy balance in part via activation of the SNS [71], regulating body weight and temperature [72-74]; chemical sympathectomy with guanethidine blocks this leptin effect, preventing diet-induced thermogenesis [75]. Leptin regulates functionally divergent hypothalamic MC4R-containing pathways to control food intake and energy expenditure [76]. MC4R mRNA has also been localized in sympathetic nervous system outflow neurons that innervate WAT [2]. How the CNS and sympathetic outflow circuits coordinately regulate lipid mobilization in WAT and thermogenesis in BAT in response to cold stress and/or diet is not fully understood, but retrograde tract tracing with pseudorabies virus suggests that MC4R-expressing cells in the hypothalamic PVN are positioned to increase sympathetic drive to both WAT and BAT [2]. It is likely, however, that both melanocortin and melanocortin-independent pathways are involved, as MC4R-deficient mice are still able to maintain normal body temperature and thermo-regulate during extended cold exposure [77], but are apparently unable to up-regulate UCP1 in BAT in response to short term cold stress or a high fat diet [3]. VGF ablation blocks obesity, hyperglycemia and hyperinsulinemia in MC4R-deficient mice [18], so this protein may function in these outflow pathways to regulate sympathetic nervous system activity and lipid storage.

Adaptive thermogenesis is mediated primarily via beta-adrenergic receptors, and genetic association studies have revealed that the genes encoding these receptors include a large number of coding and non-coding polymorphisms, which have clear functional consequences but less clear contributions to the pathogenesis of obesity, diabetes and hypertension [78]. Mice that lack β1, β2 and β3 adrenergic receptors ('beta-less' mice) are obese, primarily the result of defective SNS-dependent activation of diet-induced thermogenesis because lipolytic responses to fasting were not altered [79,80]. Siberian hamsters have an increase in sympathetic drive during short day exposure, which reduces fat mass [81,82]. This increase in SNS activity correlates with an increase in β3 receptor mRNA levels in adipose tissues [82], and interestingly, we noted an increase in β3 receptor mRNA levels in VGF knockout BAT but not WAT, and increased β2 receptor mRNA levels in WAT, perhaps correlating with increased SNS activity [79]. Recent studies indicate that norepinephrine can induce lipolysis in WAT in beta-less mice [83], suggesting a beta-adrenoreceptor independent pathway, and moreover, in the absence of norepinephrine or epinephrine, dopamine-beta-hydroxylase (DBH) knockout mice are not predisposed to obesity, as are beta-less mice, and maintain glucose tolerance [84]. The contribution of beta-adrenoreceptors and SNS activity to fat mass is therefore complicated, and notably, leptin-induced decreases in fat mass are not consistently accompanied by increases in norepinephrine (NE) turnover, a measure of SNS activity [65]. Further studies are needed to investigate whether increased HSL gene expression and lipolysis are associated with increased NE turnover in specific fat depots from VGF mutant mice.

Of note, the neuropeptides α-MSH and NPY have been demonstrated to increase and decrease lipolysis, respectively, in fully differentiated 3T3-L1 adipocytes [42], so by analogy, direct effects of VGF peptides on adipocytes are also possible, particularly in light of the expression of VGF in BAT (Figure 5) and in the sympathetic nervous system [85,86]. We quantified AQEE immunoreactive peptides by RIA in BAT, and noted that levels decrease in response to a high fat diet (HFD). Parallel studies with larger cohorts of C57Bl6 mice have revealed that C57Bl6 mice consuming a HFD compared to a chow diet generally increase their energy intake (food consumption in grams is similar) [87], and after 2 weeks, body weight has not changed [87], or has increased by ~10% ([88]; J. Mastaitis and C.V. Mobbs, personal communication and [89]). This alteration in energy balance is sufficient to trigger compensatory changes in gene expression in liver [87], including increased expression of genes involved in fatty acid oxidation. Our data would suggest that VGF C-terminal peptide TLQP62, hypothesized to decrease sympathetic outflow pathway activity to adipose tissues [17,18], is present in BAT and levels decrease as part of the compensatory response to increased energy intake.

Recent experiments have found that 14-day intracerebroventricular (icv) administration of the C-terminal VGF-derived peptide TLQP21 (VGF residues 556-576) increases energy expenditure and prevents diet-induced obesity in mice [90], and decreases food intake and body weight in hamsters [91]. Analysis of TLQP21-treated mice revealed a phenotype with numerous additional similarities to VGF knockout mice including increased energy expenditure, increased WAT β2-adrenergic receptor mRNA levels, increased WAT UCP1 mRNA levels, normal locomotor activity, and decreased WAT weight {see Results and [21]}. However, rather than very similar phenotypes, one would anticipate that mice lacking VGF and those chronically treated with VGF-derived TLQP21 peptide would potentially have opposing metabolic phenotypes. Because the precise levels of TLQP21 in brain tissue are not yet known compared to the relatively abundant C-terminal TLQP62 peptide (VGF residues 556-617), one of the major VGF-derived peptides detected in neuronal and endocrine tissues [38,92,93], and different C-terminal peptides derived from TLQP62 have opposing effects on energy balance [94], several alternative mechanisms of action are possible: (1) TLQP21 and TLQP62 could each activate its own receptor with the overall biological effect dependent on the concentration of each peptide and the distribution and affinity of putative receptors, (2) TLQP21 could inhibit actions of full length VGF or longer VGF-derived C-terminal peptides, including TLQP62 and NAPP129, which all contain the TLQP21 sequence, and/or (3) chronic TLQP21 treatment could lead to down-regulation of VGF receptor(s) and signaling. Consistent with mechanism 2, we have recently noted that TLQP21, but not a scrambled peptide, blocks TLQP62-induced electrical potentiation in hippocampal slices [95]. In addition, Severini et al. [96] have recently described a role for TLQP21 in cerebellar granule neuron survival and cellular signaling, and in the regulation of gastric motor function [97]. Taken together, these data indicate that specific VGF-derived peptides are biologically active, and may signal through independent or shared cell surface receptor(s) to regulate autonomic homeostasis and peripheral adiposity.

## Conclusion

Study of two independent VGF knockout mouse lines indicates that *Vgf *gene products regulate fat storage and energy expenditure through a mechanism that is independent of an effect on locomotor activity. Histological and electron microscopic analysis, coupled with real-time PCR and western analysis, demonstrate physiological and morphological changes in VGF germline knockout mice that are consistent with decreased fat storage, decreased adipocyte area, increased lipolysis, and decreased lipogenesis in WAT, and increased fatty acid oxidation, increased UCP1 and UCP2 protein levels, and increased mitochondrial number and cristae density in BAT. Identification of VGF in BAT, together with these data, supports the hypothesis that VGF and/or VGF-derived peptides modulate sympathetic outflow pathways that regulate adiposity and energy expenditure.

## Methods

### Mouse Strains and Diets

Previously described mice with a targeted deletion of the *Vgf *gene on a mixed C57Bl/6 and 129/SVJ background (designated VGF_M _or *Vgf*_*M *_*-/-*) [21], were repetitively backcrossed 10 generations onto wild type C57Bl/6 mice (designated VGF_C57 _or *Vgf*_*C*57 _*-/-)*, and heterozygous *Vgf+/Vgf- *mice on a homogenous C57Bl/6 background were mated to generate wild type, heterozygous, and homozygous VGF-deficient mice. A second VGF-deficient line was generated by Regeneron Pharmaceuticals Inc. as previously described [25] using F1H4 ES cells (a 129B6/F1-derived cell line) and a BAC-based targeting vector with deletion of the entire *Vgf *coding sequence and insertion of an in frame *lacZ *reporter gene and neomycin-selection cassette; chimeric mice resulted from the injection of two independent *Vgf-/Vgf- *embryonic stem cell clones into C57BL6/J blastocysts. Male chimeras were mated with C57BL6/J females to produce F1 breeders and experiments were performed on N2F1 mice (>83% C57Bl6 background; designated VGF_R _or *Vgf*_*R *_*-/-*) from two separate clones with similar results.

Mice were housed at room temperature in a 12 h light, 12 h dark cycle, with chow and water available adlib unless otherwise specified. Mice fed standard chow received a 4.5% fat, 55% carbohydrate, 20% protein, 4.7% fiber diet (Purina PicoLab Rodent Diet 20-5053; 4 Kcal/gm; Purina. St. Louis, MO). For high fat diet studies, mice received a high fat (33.5-35.5%) and high simple carbohydrate (34-35.5%) diet that also contained 20% protein and 0.1% fiber (Bio-Serv F2685; 5.4 kcal/g). All animal studies were conducted in accordance with the Guide for Care and Use of Experimental Animals, using protocols approved by Institutional Animal Care and Use Committees at Mount Sinai School of Medicine or Regeneron Pharmaceuticals Inc.

### Tissue Preparation and Immunohistochemistry

Mice were sacrificed and white and brown adipose tissues removed, fixed in formalin and embedded in paraffin. Sections (5 μm) were stained with hemotoxylin and eosin. Transcardial perfusions were performed with 4% paraformaldehyde in PBS after washing in saline solution. After perfusion brown adipose tissue was dissected and fixed overnight in the same fixative and then embedded in paraffin for immunohistochemistry. Polyclonal rabbit antibodies were raised against a VGF (amino acids 8-349)-trpE fusion protein [98]. Dewaxed sections (10 μm) were processed through the following steps for antigen retrieval: (1) treated with 0.3% hydrogen peroxide in methanol for 20 minutes; (2) microwaved for 20 minutes in 10 mM citric acid buffer to retrieve antigens; (3) incubated for 48 hours at 4°C in PBS that contained 0.3% Triton X100, 3% normal goat, 3% horse serum, and anti-VGF or anti-tyrosine hydroxylase (Chemicon International, Temecula, CA) antibody (1:50); (4) incubated for 1 hour in biotinylated secondary antibody, goat anti-rabbit IgG (1:200). Staining was performed using an avidin:biotyinylated enzyme complex (vector ABC kit, Vector laboratories, Burlingame, CA) with diaminobenzidine as a substrate. For immunofluorescence, polyclonal rabbit anti-VGF (1:1000) was used to stain BAT. Dewaxed sections (10 μm) were subjected to an antigen retrieval step, were incubated overnight at 4°C in PBS that contained 0.3% Triton X100, 3% normal goat, 3% horse serum, and VGF antibody (1:50), and lastly with Texas red goat anti-rabbit 1:250 for 1 hour. Staining was visualized using a Zeiss Axiophot microscope.

### Morphometry

Isolated BAT and WAT were immersed and fixed in formalin, embedded in paraffin, sectioned, and stained with H&E. Slides were analyzed using Zeiss Axiophot microscope and images captured with a SPOT digital camera. Calculation of mean cell size, lipid area, and cell size distribution was performed using MetaMorph Imaging Software (Molecular Devices, Downingtown, PA). For image analysis, 6 images per group were randomly selected to measure the mean cell size and size distribution in WAT. Groups consisted on 2 mice of each genotype [wild type (*Vgf+/Vgf+*), heterozygous knockout (*Vgf+/Vgf-*), and homozygous knockout (*Vgf-/Vgf-*)], and images were taken with 20× and 10× objectives for BAT and WAT, respectively. Mean cell size and size distribution were determined from 390 adipocytes in WAT. As it was difficult to outline the cells in BAT because of the accumulation of lipid in the cytoplasm, lipid area in BAT was quantified using MetaMorph Imaging Software.

### Electron Microscopy

For electron microscopic examination, BAT samples were placed in ice-cold fixative buffer (3% gluteraldehyde in 0.2 M sodium cacodylate buffer pH 7.3) for 2 h. Specimens were washed in 0.2 M sodium cacodylate buffer pH 7.3 for 10 min, postfixed in 1% osmium tetroxide in 0.2 M sodium cacodylate buffer for 1 h, washed and dehydrated in graded ethyl alcohol steps, and embedded in pure EPON A&B mixture with DMP-30. Ultrathin sections for electron microscopy were examined using a Hitachi H7650 instrument linked to an SIA digital camera (Scientific Instruments and Applications, Duluth, GA) controlled by Maxim CCD software. Electron micrographs were obtained at 20,000× magnification, and mitochondrial analysis was carried out using MetaMorph Imaging Software. Six images per group [2 mice of each genotype: wild type (*Vgf+/Vgf+*) and homozygous knockout (*Vgf-/Vgf-*)] were randomly chosen, and the number and the area of mitochondria/μm^2 ^were measured. The mean mitochondrial area/μm^2 ^was determined from 316 mitochondria in BAT. The cristea number was calculated from 40 mitochondria for each group (2 images/group).

### Western Blot Analysis

BAT and epididymal WAT from wild type (*Vgf+/Vgf+*), heterozygous knockout (*Vgf+/Vgf-*), and homozygous knockout (*Vgf-/Vgf-*) (n = 5 mice for each group) were homogenized in ice cold lysis buffer [50 mM Tris-HCl (pH 8) 150 mM NaCl, 0.02% sodium azide, 0.1% SDS, 0.5% deoxycholate and 1% NP40] supplemented with protease and phosphatase inhibitor cocktails (Roche Diagnostics, Mannheim, Germany; Thermo Scientific, Waltham, MA). Lysates were cleared by centrifugation (14,000 rpm, 10 min at 4°C), and the protein concentration of the supernatant determined by the BCA protein method (Thermo Scientific). Protein samples (25 μg) were separated by SDS-PAGE and transferred to nitrocellulose membranes (Millipore, Bedford, MA). Membranes were blocked with 5% (W/V) BSA in phosphate-buffered saline, and were probed with rabbit polyconal anti-UCP1 [1:1000 (v/v)] (Calbiochem, San Diego CA), rabbit polyconal anti-UCP2 [1:1000 (v/v)] (Calbiochem), or mouse monoclonal anti-GAPDH [1:1000 (v/v)] (Cell Signaling Technology, Danvers, MA), at 4°C overnight. After 3 washes in PBS-T (0.1% Tween in PBS) membranes were incubated for 1 hour with HRP-conjugated secondary antibody [1:10,000 (v/v) in PBS-T] (GE Healthcare, Piscataway, NJ) containing 5% non-fat dry milk. After 3 washes in PBS-T bound antibodies were detected using ECL (Thermo Scientific) and exposure to HyBlot CL (Denville Scientific, Metuchen, NJ). Densitometric quantification was performed using NIH ImageJ.

### β-galactosidase Histology

For β-galactosidase staining, mice were deeply anesthetized (240 mg/kg ketamine, 48 mg/kg xylazine i.m.) and exsanguinated with ice-cold heparinized saline. Tissues were fixed by transcardial perfusion of 2% paraformaldehyde in 0.1 M phosphate buffer, postfixed for 2 hr, and then washed in several changes of phosphate-buffered saline. Whole mount staining was then carried out essentially as previously described [99,100], preparations were examined, and then cryoprotected for a least 24 hr in two changes of buffered 30% sucrose at 4°C with agitation before sectioning.

### Radioimmunoassay

Tissues were sonicated and extracted in 0.1 M acetic acid for 6-7 min at 70°C [101]. Homogenates were subjected to centrifugation (14,000 × g for 30 min, 4°C), and the supernatants were dried using a Speed Vac concentrator (Sorvall) and stored at -80°C. Samples were resuspended in 0.1 M NaHPO_4_, 0.1% TritonX-100, pH 7.2 and subjected to RIA. Rabbit polyclonal antiserum directed against the VGF-derived C-terminal AQEE30 peptide was utilized, and RIAs carried out as previously described [38]. Under these acid extraction conditions, the RIA directed against the C-terminal AQEE30 peptide would also be expected to cross-react with N-terminally extended peptides TLQP62 and NAPP129 in these tissue extracts.

### RNA isolation and RT-PCR

Mice were sacrificed, and brown and white (epididymal fat pads) adipose tissues were removed, flash frozen on dry ice, and stored at -80°C until use. Fat pads were homogenized in Trizol and RNA was extracted according to the manufacturer's instructions (Invitrogen Corporation, Carlsbad, CA). Total RNA (5 μg) was reverse transcribed in a 20 μl reaction using Superscript II enzyme (Invitrogen Corporation, Carlsbad, CA), and single stranded cDNA was diluted 1:50 for RT-PCR. Using Syber Green Master Mix (Applied Biosystems, Foster City, CA), amplification was performed in ABI Prism 8500 system for 40 cycles in quadruplicate. SDS 2.1 software was used for analyzing cycle threshold (C_t_) values, and expression of the housekeeping gene beta actin was used to normalize levels according to the following formula: ΔΔCt = (C_t _geneX *VGFko *- C_t _actin *VGFko*) - (C_t _geneX *wild type *- C_t _actin *wild type*). Fold difference in mRNA levels between *Vgf-/Vgf- *and *Vgf+/Vgf+ *= 2^ΔΔCt^. Forward and reverse primers were designed based on published studies and on database analysis as follows: acetyl-CoA carboxylase (ACC), 5'-CAGATCCAGGCCATGTTGAGACG and 3'-TCGCTGGGTGGGTGAGATGTG; beta-1-adrenergic receptor (β1AR), 5'-CGGCTGCAGACGCTCACCAA and 3'-CGCCACCAGTGCATGAGGAT; beta-2-adrenergic receptor (β2AR), 5'-GCTGCAGAAGATAGACAAAT and 3'-GGGATCCTCACACAGCAGTT; beta-3-adrenergic receptor (β3AR), 5'-CTGCTAGCATCGAGACCTT and 3'-CGAGCATAGACGAAGAGCAT; carnitine palmitoyltransferase 1 (CPT1), 5'-ACCACTGGCCGAATGTCACAA and 3'-AGCGCGTAGCGCATGGTCAT; cytochrome C oxidase II (COX II), 5'-CGATTCCAACTTGGTCTACAA and 3'-GGAACCATTTCTAGGACAATG; diacylglycerol acyltranferase (DGAT), 5'-TCCGCCTCTGGGCATTC and 3'-GAATCGGCCCACAATCCA; fatty acid synthase (FAS), 5'-TGCTCCCAGCTGCAGGC and 3'-GCCCGGTAGCTCTGGGTGTA; glucose-4-transporter (GLUT4), 5'-CTCAGCAGCGAGTGACTGGGAC and 3'-CCCTGAGTAGGCGCCAATGAGG; glycerol-3-phosphate acyltransferase (GPAT), 5'-CAGCTCTGCTGCCATCTTTG and 3'-TGCAGCTTCTGCAGGTACTCA; hormone sensitive lipase (HSL), 5'-GAAAAACAGCCTGTCGGACCA and 3'-CCAGGGCGATCTGCAGGT; insulin receptor (IR), 5'-GTAGCCTGATCATCAACATCCG and 3'-CCTGCCCATCAAACTCTGTCAC; insulin receptor substrate 1 (IRS1), 5'-CCTCTCCAACGCCAGAAGCTGCC and 3'-ATGGCGAGCCCTCCGGATACCG; insulin receptor substrate 2 (IRS2), 5'-GGATAATGGTGACTATACCGAGA and 3'-CTCACATCGATGGCGATATAGTT; lipoprotein lipase (LPL), 5'-AGGACCCCTGAAGACAC and 3'-GGCACCCAACTCTCATA; peroxisome proliferator activated receptor alpha (PPARα), 5'-TCCCTTGTAGCCTTTTGTCAT and 3'-AAGCCATTGCCGTACGCGAT; peroxisome proliferator activated receptor gamma (PPARγ), 5'-AGGCCGAGAAGGAGAAGCTGTTG and 3'-TGGCCACCTCTTTGCTCTGCTC; PPARγ coactivator 1 (PGC-1), 5'-AAAGAATTCGAACTAAGGGATGGCGACTT and 3'-ATAGGATCCGGAATATGGTGATCGGGAAC; sterol regulatory element binding protein 1C (SREBP1C), 5'-GAGGCACTCCCCCAAAAGAT and 3'-TGATGAGAGGGAGGCCATTT; uncoupling protein 1 (UCP1), 5'-GATCCAAGGTGAAGGCCAGG and 3'-GTTGACAAGCTTTCTGTGG; uncoupling protein 2 (UCP2), 5'-GTGACCTGCTGCGCTGTGGTACT and 3'-GATCCAAGGGGAGAGTCA; housekeeping gene (beta actin), 5'-TGCTGTCCCTGTATGCCTCT and 3'-AGGTCTTTACGGATGTCACG

### Indirect Calorimetry, Body Composition and Serum Chemistries

Metabolic measurements were assessed on a regular diet (at 9-10 weeks of age) and were obtained using an Oxymax (Columbus Instruments International Corp.) open circuit indirect calorimetry system as previously described [102]. The first 2 h was a period of adaptation for the animals, and metabolic rate (*V*O_2_), RQ (ratio of *V*CO_2_/*V*O_2_) and activity (counts) were then evaluated for a 72-h period. Energy expenditure was calculated as the product of calorific value of oxygen (calorific value of oxygen = 3.815 + 1.232 RQ) and the volume of O_2 _consumed and this was normalized for body weight in kilograms. Two cohorts of male mice between 2-3 months of age (n = 7-8 mice of each genotype per cohort) were utilized for body composition, serum chemistry and metabolic cage analysis, carried out as described above and detailed previously [103].

### Data and Statistical Analysis

Data are expressed as mean ± SEM. Comparisons were performed using *t *test or repeated-measures analysis of variance (ANOVA) where appropriate, using the programs Statview and Prism. P-values less than 0.05 were considered significant. For metabolic cage analysis, basal metabolic rate (BMR), defined as oxygen consumption during the light rest period, was determined by comparing VO_2 _measurements of VGF_R _knockout and wild-type littermates by two-tailed *t*-test. For real-time RT-PCR, assays were carried out in quadruplicate, and mean cycle threshold (C_t_) values (± SEM) were compared by two-way ANOVA (p ≤ 0.05 was considered significant). These values were used to calculate fold difference in mRNA levels (2^ΔΔCt^).

## Authors' contributions

EW carried out histological analysis, metabolic studies, real-time PCR experiments, and drafted the manuscript, SF carried out histological and EM analysis, morphometry, western blot analysis, and helped to draft the manuscript; HO carried out the metabolic and feeding analysis, whole mount β-galactosidase staining, and serum analysis; MS carried out the breeding and genotyping of VGF-deficient mice and assisted with histological, EM and western analysis; REG assisted with the design and coordination of the electron microscopic analysis; TC carried out the RIA analysis; MWS participated in the design and coordination of the study; SRS participated in the design and coordination of the study, and drafted the manuscript. All authors read and approved the final manuscript.

## Supplementary Material

Additional file 1 **Supplemental Figure 1. Lipid deposition in multilocular cells of brown adipose tissue (BAT) and unilocular cells of white adipose tissue (WAT) is reduced in VGF_C57 _knockout mice**. Representative sections of interscapular BAT (A-D) and epididymal WAT (E-F) from ad lib-fed *Vgf*_*C*57 _*-/- *(A, C and E) and age-matched *Vgf*_*C*57 _*+/+ *(B, D, and F) mice were stained with H&E. The interscapular WAT that normally surrounds BAT in wild type mice (D) was absent in VGF-deficient mice (A and C). Adipocytes in interscapular BAT and epididymal WAT from *Vgf*_*C*57 _*-/- *mice (A and E) contain less lipid accumulation than the corresponding adipocytes from wild type mice (B and F). Scale bars are 100 μm in length. Click here for file

Additional file 2 **Supplemental Figure 2. Neonatal chemical sympathectomy with guanethidine (GE) increases fat and glycogen stores and plasma glucose levels in GE-treated VGF knockout mice**. H&E staining of liver, BAT, and epididymal WAT from 4 month-old *Vgf*_*C*57 _*-/- *mice treated daily with GE (50 mg/kg) or saline (control) from postnatal day 5 (P5) until P25, essentially as previously described [104,105], which results in permanent sympathectomy. At 4 months of age, mice were anesthetized, blood samples collected, and glucose levels determined by glucometer; tissues were removed and formalin-fixed for histological analysis. Note that clear areas in liver, corresponding to glycogen deposits, are increased in GE-treated *Vgf*_*C*57 _*-/- *mice compared to saline-treated control *Vgf*_*C*57 _*-/- *mice (panels A and B). BAT in GE-treated *Vgf*_*C*57 _*-/- *mice is bordered by WAT (arrows), which is reduced in saline-treated *Vgf*_*C*57 _*-/- *mice (panels C and D). Fat storage is reduced in WAT from saline-treated compared to GE-treated *Vgf*_*C*57 _*-/- *mice (panels E and F). Scale bars are 100 μm in length. GE-treatment significantly increased circulating glucose levels in *Vgf*_*C*57 _*-/- *but not in *Vgf*_*C*57 _*+/- *mice compared to their respective saline-treated controls (panel G; ANOVA, p < 0.05, mean ± SEM). Click here for file

Additional file 3 **Supplemental Figure 3. Immunohistochemical localization of VGF protein in BAT**. Immunohistochemical staining of BAT with anti-VGF and anti-tyrosine hydroxylase (TH) antisera in wild-type mice was carried out, and showed similar patterns. Sections were stained with rabbit anti-VGF_78-340 _[98] (panels A, C, and D) and anti-TH (Chemicon International, Temecula, CA) (panel B) (in panels A and B, arrows point to VGF positive and TH positive fibers, respectively). Note that VGF immunoreactivity is also seen in the adventitia of small arterioles (panel C; arrows). In addition, VGF-positive puncta in direct contact with the brown adipocyte (panel D) were visualized using immunofluorescent staining. Scale bars are 100 μm (panels A-C) and 10 μm (panel D). Click here for file
