# Supplementary figures and images for: Analysis of knockout mice suggests a role for VGF in the control of fat storage and energy expenditure
Source: BMC Physiol. 2009 Oct 28;9:19. doi: 10.1186/1472-6793-9-19 (PMC2774661; doi:10.1186/1472-6793-9-19)

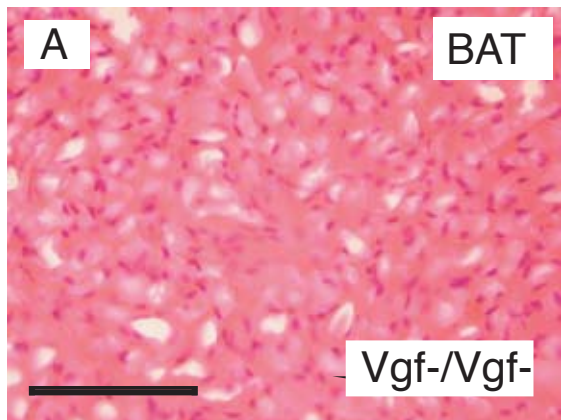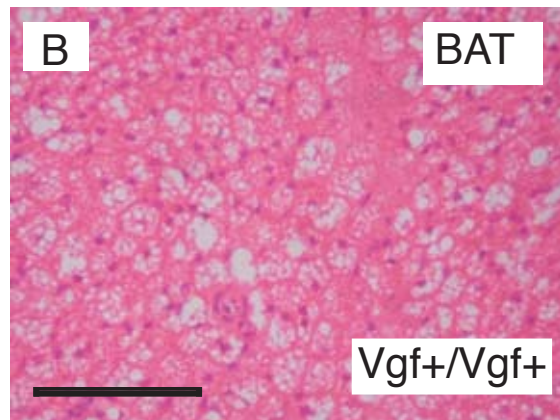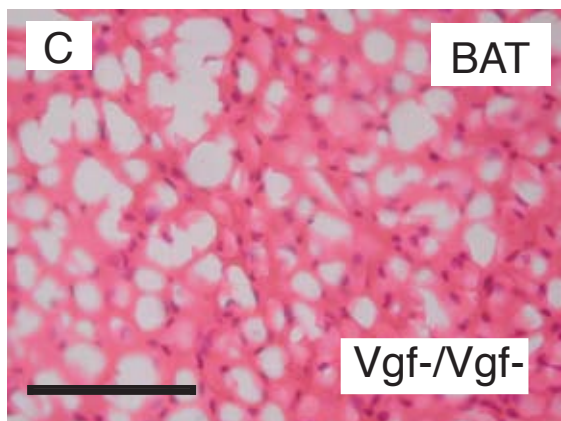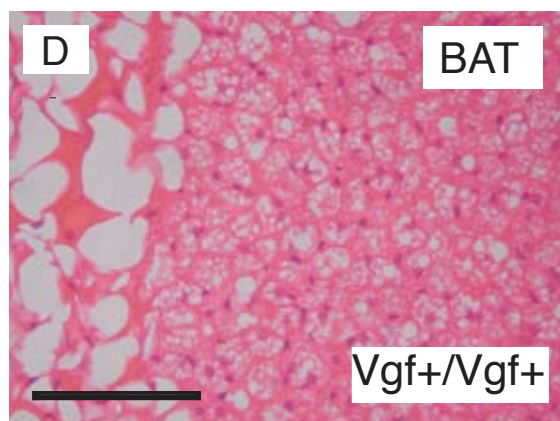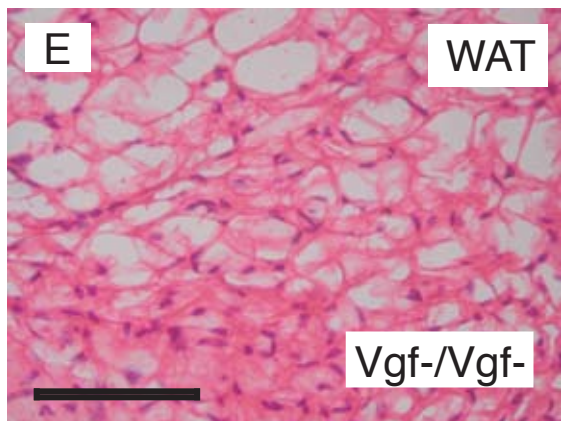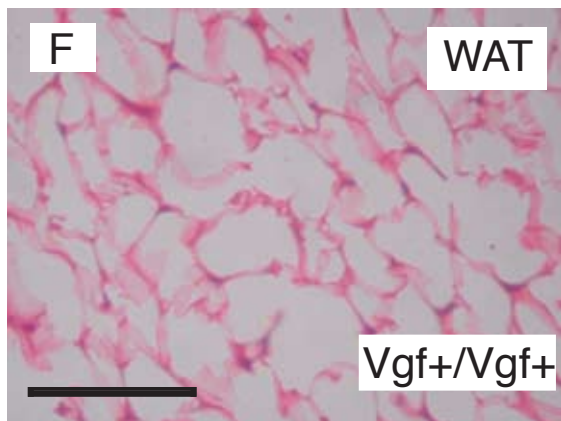

Supplement: Additional file 1 — Supplemental Figure 1. Lipid deposition in multilocular cells of brown adipose tissue (BAT) and unilocular cells of white adipose tissue (WAT) is reduced in VGFC57 knockout mice. Representative sections of interscapular BAT (A-D) and epididymal WAT (E-F) from ad lib-fed VgfC57 -/- (A, C and E) and age-matched VgfC57 +/+ (B, D, and F) mice were stained with H&E. The interscapular WAT that normally surrounds BAT in wild type mice (D) was absent in VGF-deficient mice (A and C). Adipocytes in interscapular BAT and epididymal WAT from VgfC57 -/- mice (A and E) contain less lipid accumulation than the corresponding adipocytes from wild type mice (B and F). Scale bars are 100 μm in length. [file 1472-6793-9-19-S1.pdf]

## Control

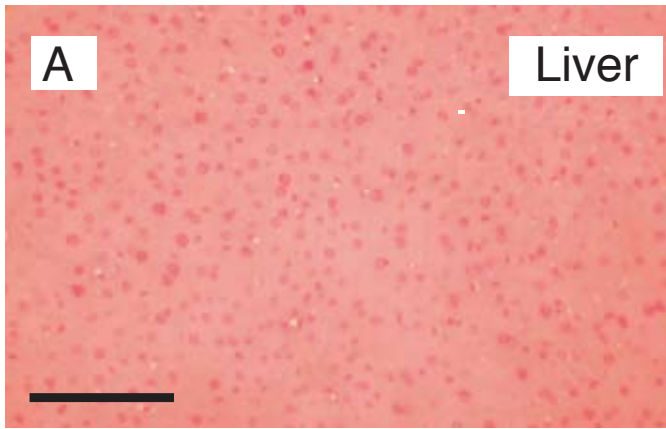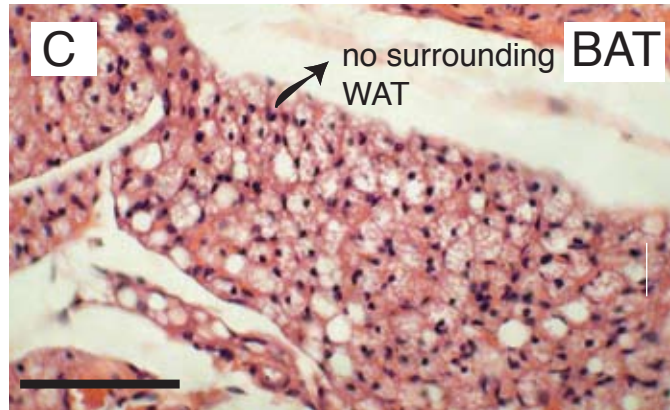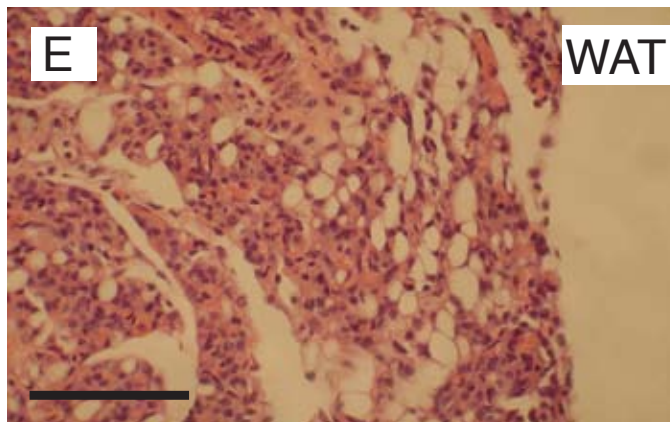

## GE-Treated

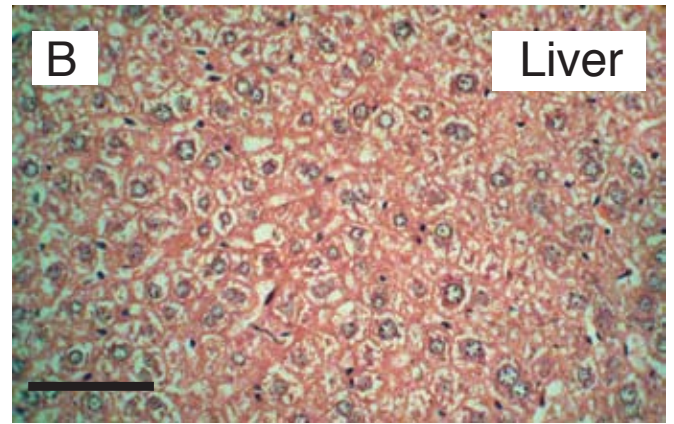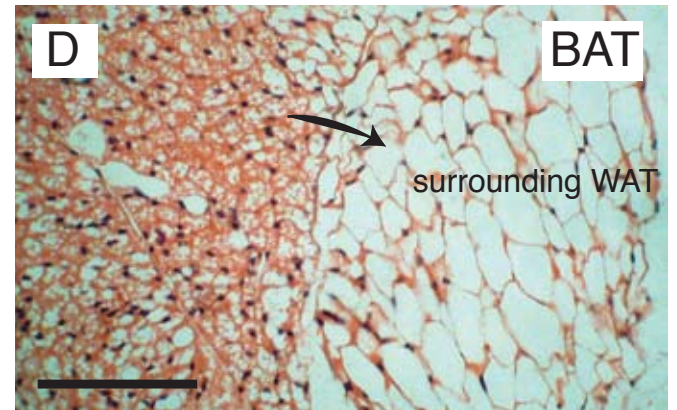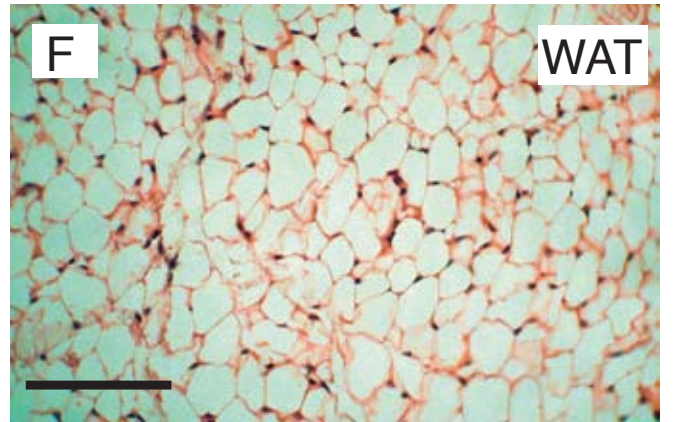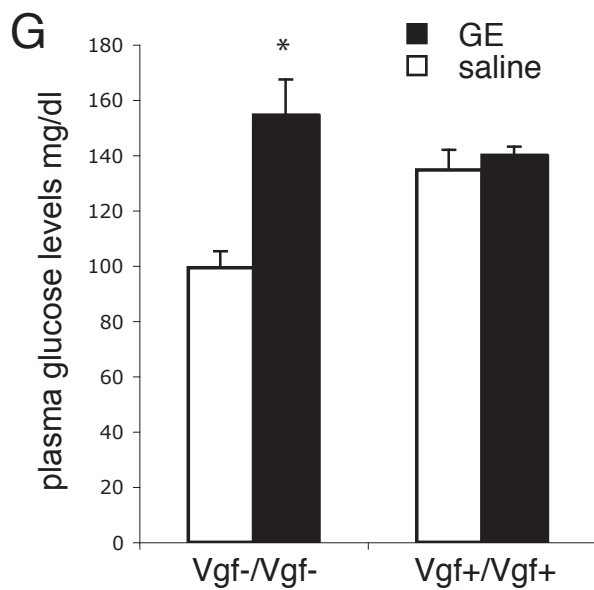

Supplement: Additional file 2 — Supplemental Figure 2. Neonatal chemical sympathectomy with guanethidine (GE) increases fat and glycogen stores and plasma glucose levels in GE-treated VGF knockout mice. H&E staining of liver, BAT, and epididymal WAT from 4 month-old VgfC57 -/- mice treated daily with GE (50 mg/kg) or saline (control) from postnatal day 5 (P5) until P25, essentially as previously described [104,105], which results in permanent sympathectomy. At 4 months of age, mice were anesthetized, blood samples collected, and glucose levels determined by glucometer; tissues were removed and formalin-fixed for histological analysis. Note that clear areas in liver, corresponding to glycogen deposits, are increased in GE-treated VgfC57 -/- mice compared to saline-treated control VgfC57 -/- mice (panels A and B). BAT in GE-treated VgfC57 -/- mice is bordered by WAT (arrows), which is reduced in saline-treated VgfC57 -/- mice (panels C and D). Fat storage is reduced in WAT from saline-treated compared to GE-treated VgfC57 -/- mice (panels E and F). Scale bars are 100 μm in length. GE-treatment significantly increased circulating glucose levels in VgfC57 -/- but not in VgfC57 +/- mice compared to their respective saline-treated controls (panel G; ANOVA, p < 0.05, mean ± SEM). [file 1472-6793-9-19-S2.pdf]

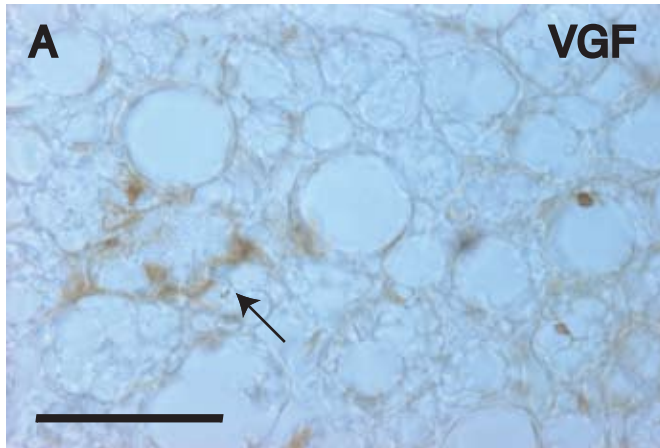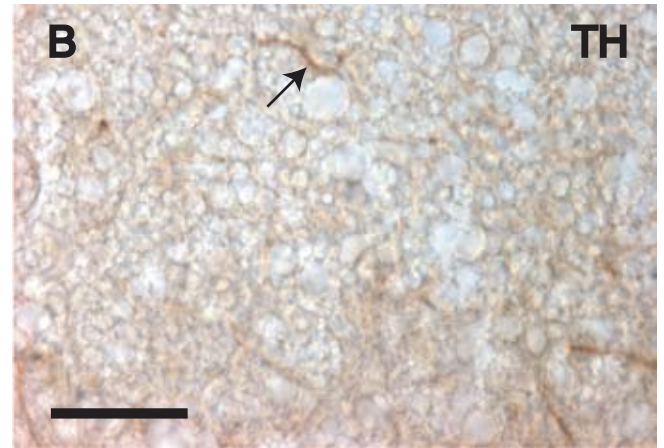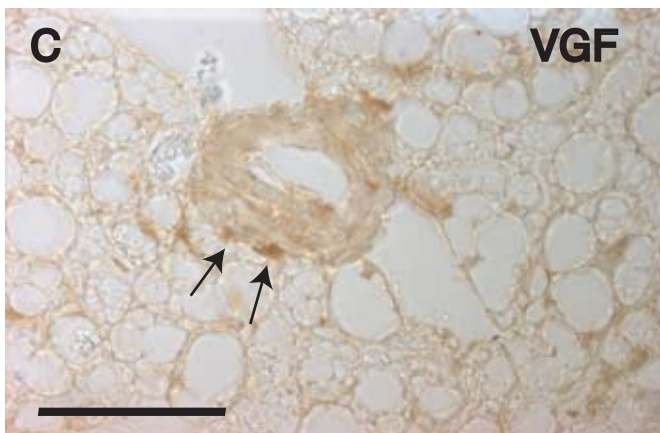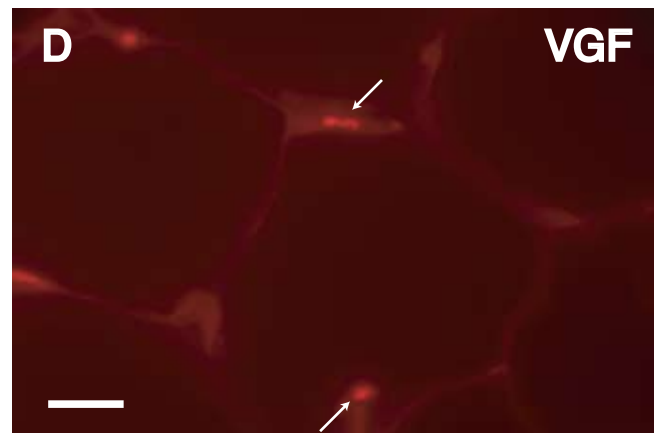

Supplement: Additional file 3 — Supplemental Figure 3. Immunohistochemical localization of VGF protein in BAT. Immunohistochemical staining of BAT with anti-VGF and anti-tyrosine hydroxylase (TH) antisera in wild-type mice was carried out, and showed similar patterns. Sections were stained with rabbit anti-VGF78-340 [98] (panels A, C, and D) and anti-TH (Chemicon International, Temecula, CA) (panel B) (in panels A and B, arrows point to VGF positive and TH positive fibers, respectively). Note that VGF immunoreactivity is also seen in the adventitia of small arterioles (panel C; arrows). In addition, VGF-positive puncta in direct contact with the brown adipocyte (panel D) were visualized using immunofluorescent staining. Scale bars are 100 μm (panels A-C) and 10 μm (panel D). [file 1472-6793-9-19-S3.pdf]
